# Evaluation of exploration time, accuracy, and task difficulty perception in three tactile tests among physiotherapy students

**DOI:** 10.1080/10669817.2025.2465729

**Published:** 2025-02-15

**Authors:** Nataša Mlakar, Sonja Hlebš

**Affiliations:** Department of Physiotherapy, Faculty of Health Sciences, University of Ljubljana, Ljubljana, Slovenia

**Keywords:** Palpation, tactile sensitivity, physical therapy, undergraduate education, postgraduate education

## Abstract

**Objectives:**

Tactile sensitivity is one of the most important skills for developing competence in manual palpation. There is a lack of studies aimed at analyzing the development of tactile sensitivity during different levels of physiotherapy education. The present study aims to compare manual tactile sensitivity in two groups of physiotherapy students.

**Methods:**

Twenty first-year physiotherapy students (mean age 19.4 yrs ± 0.6) and twenty final-year physiotherapy students (mean age 23.7 yrs ± 3.7) participated in the study. For the tactile sensitivity test, 3 wooden tables were used, in which different geometric structures were engraved. Subjects were instructed to perform a tactile examination of the geometric structures and then reproduce them by drawing on a sheet of paper. The tactile time, drawing time, accuracy, and difficulty of the geometric structures were scored. A two-sample t-test was used for the between-groups comparison if more time in an educational program should result in differences in tactile time, drawing time, accuracy, and difficulty. Linear regression was used to compare the difficulty with the accuracy of all geometric structures. Wilcoxon test was used to test the intra-rater agreement.

**Results:**

The accuracy of the reproduction of geometric structures 1, 2 and 3 were 77.5%, 27.5% and 45%, for all physiotherapy students respectively. Final-year physiotherapy students spent more time exploring geometric structure 2 (*p* = 0.014) and geometric structure 3 (*p* = 0.0018) compared to first-year physiotherapy students. No statistically significant differences were found between groups in drawing time, accuracy, and difficulty of geometric structures. The examiner showed a high intra-rater agreement in the assessment (over 96%).

**Discussion/conclusion:**

The study showed that the level of education and gained experience during laboratory teaching may be important in improving the palpation skills of physiotherapy students. Tactile sensitivity exercises should be included in physiotherapy education programs.

## Introduction

Palpation is a physical examination technique in which objects, such as organs or body parts, are palpated with the fingers to determine their size, shape, consistency, and location. Many medical procedures use palpation as a complementary interaction technique and can therefore be considered an essential basic method [1]. When humans palpate an object with their fingertips, they acquire a composite perception of its properties. In addition to texture, temperature, and temporal properties, the geometric features of an object also make an important contribution to haptic perception. The haptic perception of geometric features includes tactile, somatosensory and motor activities [2]. Tactile sensitivity refers to the skin’s ability to recognize and respond to physical stimuli such as pressure, texture, temperature and vibration. Due to a higher density of sensory receptors and smaller receptive fields, the fingertips have a higher spatial acuity and play a crucial role in haptic perception [3].

Palpation is a diagnostic method in physiotherapy to assess quality, morphological changes, and sensitivity of tissue [1]. Manual palpation is one of the components of clinical examination most commonly taught and practiced in physiotherapy curricula to identify anatomical regions; explore pain, consistency, resistance, and mechanical sensitivity of body tissues, and evaluate symmetry and quality of motion [2]. In manual palpation, the examiner actively explores two- or three-dimensional structures (haptic perception) [4,5] or palpates passively, exploring with only static contact (tactile perception) [6,7].

Simulated palpation training has been shown to be effective in improving palpation skills [8]. Some studies have reported that the level of study (undergraduate or postgraduate) has an impact on reproducibility and validity in manual palpation [9,10].

Palpation skills depend not only on previous examiner experience [1] but also on individual sensitivity, education, age, and disease [11–16]. Physiotherapists who work with patients daily have more tactile experience and are more likely to be able to distinguish between normal and abnormal structures [1]. Blind people and musicians have better tactile skills due to everyday use and experience [11]. However, tactile sensitivity is task and body part specific (e.g. hand or fingers) and not just an effect of training [17].

The teaching of palpation as part of physiotherapy education should enable students to practically describe what they feel and provide a safe, non-threatening, supportive and non-judgmental environment [18]. Students should be able to palpate different individuals, link palpation to anatomical knowledge and demonstrate progress in their palpation skills [18]. Familiarization with the literature is correlated to confidence performing palpation in manual therapists [19]. Teaching a therapeutic psychomotor skill to students should be done in four steps: 1) demonstration of the skill by the instructor during the lab session, 2) students practicing together during the lab session, 3) individual practice at home using a handout and videotape, and 4) feedback on the performance of the skill by an instructor acting as a patient [20]. Moreover, the class influences how an individual student learns [18] and body painting for palpation accuracy in novice palpators can be an enjoyable and entertaining activity [21].

Palpation is not only difficult to develop and teach, but also to assess [22]. Tactile sensitivity tests can be used as a tool to train and assess tactile sensitivity in physiotherapy students. Low-cost and easy-to-make test instruments could be useful assessment tools for palpation training [23,24]. However, a sheet of paper or a wooden relief does not represent a human tissue, but the qualitative and quantitative information from a single assessment can be used to promote learning [23,25,26]. Such assessment tools can provide a benchmark to guide the learner who is approaching a relatively unstructured body of knowledge [27]. Various authors report the use of solid materials such as coins, cork or wood when teaching palpation [24,28,29]. These structures can help a student in the initial stages of learning palpation to understand the purpose of palpation and the meaning of touch [28]. Previous authors have reported the use of a sunken relief structure for learning or testing tactile sensitivity in healthy individuals [12], anorexia nervosa [12], physiotherapy students [30] and female physiotherapy students [31]. Palpating the edges of a figure and drawing its shape can be a teaching method for edge discrimination. Edge discrimination is important for palpating bony landmarks, such as the edges of bones like the scapula or the iliac crest [24].

It seems plausible that tactile sensitivity increases with training during education, yet there is little research on the influence of educational level on tactile sensitivity in healthcare students. Therefore, the aim of our study was to compare the manual tactile sensitivity in two groups of physiotherapy students (first-year physiotherapy and final-year physiotherapy students) in terms of the evaluation of the exploration time (time to palpate the structure), accuracy (how accurate a participant reproduces palpated structure by drawing on a sheet of paper) and perception of task difficulty in three tactile perception tests (rating the difficulty of the task on a Likert scale of 1–5).

## Methods

### Participants

Participants were physiotherapy students recruited from the Faculty of Health Sciences Ljubljana (Slovenia). Potential subjects were invited to participate through a written invitation posted on the bulletin board. If subjects expressed interest in participating, further verbal information was provided, and questions were answered in an individual interview. Information was given by a member of the research team; teachers were not involved in recruitment to avoid any coercion on students. Written information was also provided. Inclusion criteria were set according to the study objectives: 1) first-year physiotherapy students (*n* = 75) and 2) final-year physiotherapy students (*n* = 89). None of the participants suffered from any upper extremity injury in the last six months prior to the study. Forty subjects of those who expresses interest met the inclusion criteria and voluntarily participated in the study (first-year physiotherapy students were undergraduate students, *n* = 20, mean age 19.4 years ± 0.6; final-year physiotherapy students were students from third (last) year of undergraduate study and second (last) year of master study, *n* = 20, mean age 23.7 years ± 3.7). Participants signed an informed consent form agreeing to participate in the study. The study protocol was approved by the National Medical Ethics Committee of the Republic of Slovenia (0120–17/2019/4).

### Procedure

**Figure 1.**
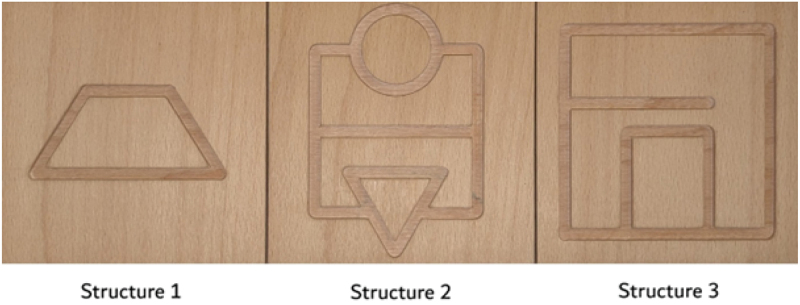
Geometric relief structures 1, 2 and 3 (Photo credit: Nataša Mlakar).

Following the idea of Grunwald et al. [12] and Nascimento et al. [30], three two dimensional (13 cm x 13 cm), wooden, square structures with geometrically sunken reliefs (7 mm width, 3 mm depth) were used to assess palpation skills (Figure 1). The geometric relief structures provided different stimulus complexity. This test has already been used in the investigation of tactile sensitivity in healthy [30] and pathological conditions [12].

Participants were allowed to familiarize themselves with the geometric relief structure before the experiment by palpating a sample not included in the experiment and practicing tactile examination for 1 min with both hands with eyes open, as described by Grunwald et al. [12].

**Figure 2.**
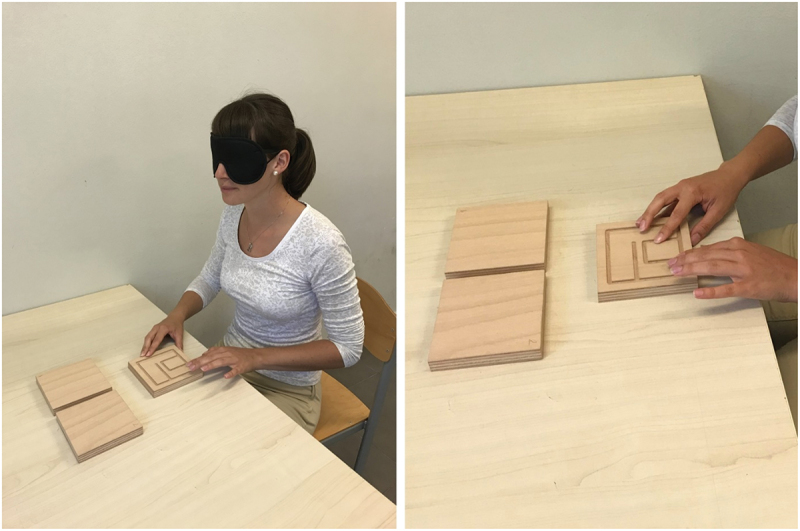
Testing procedure – tactile examination (Photo credit: Nataša Mlakar).

The task consisted of exploring three geometric structures, that were presented to the participants in the random order. They were instructed to palpate the structure with both hands while keeping their eyes closed and covered (Figure 2). Participants were informed that they had 3 minutes to complete the task and were given feedback on the remaining time at 1 min, 2 min, 2 min 30 s, and 2 min 50 s, according to Nascimento et al. [30].

**Figure 3.**
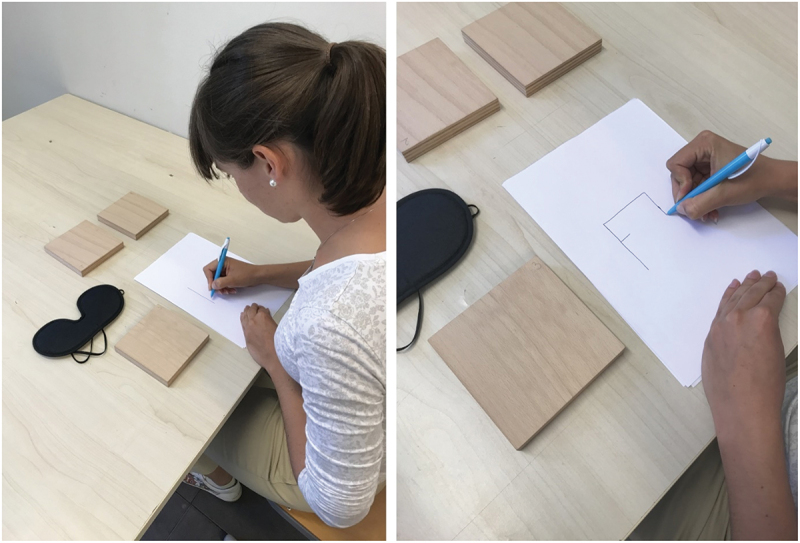
Testing procedure – reproduction of structure (Photo credit: Nataša Mlakar).

After palpating the geometric structures, all participants were asked to reproduce the geometric structure as accurately as possible by drawing on a sheet of paper with their eyes open (Figure 3). There was no time limit for reproducing the geometric structure. Participants were not given feedback on the accuracy of the reproduction.

The time spent palpating each geometric structure and the time spent drawing each reproduction were measured (using a stopwatch). After completing each reproduction, participants were asked to rate the difficulty of the task on a Likert scale of 1–5, where 1 was extremely easy, 2 easy, 3 not easy and not difficult, 4 difficult, and 5 extremely difficult.

The evaluation of the reproduction of the geometric structures was graded from 1 to 4 according to Grunwald et al. [12] (Table 1). Intra-rater agreement was determined by having all drawings evaluated twice by the same examiner, with the second assessment of the same drawings (grade b) taking place one week after the first assessment (grade a).

**Table 1.** The evaluation of the reproduction of geometric structures.

Grade	
1	Correct reproduction of the structure.
2	Correct reproduction of the structure with one to three errors.
3	Inadequate reproduction of the structure, only correct reproduction of individual elements.
4	Incorrect reproduction of the structure or individual elements.

### Statistical analysis

All statistical analyses were conducted using Python version 3.8, Python Software Foundation, Wilmington, Delaware, United States. A two-samples t-test was used for between groups comparison if more time in an educational program should result in differences in tactile time, drawing time, accuracy, and difficulty; linear regression was used to compare difficulty with accuracy in drawing all geometric structures. The Wilcoxon test was used to test intra-rater agreement. Alpha was set at 0.05.

## Results

Both groups of physiotherapy students showed high accuracy in the reproduction of the geometric structure 1, in 77.5% the reproduction was correct. The accuracy of the reproduction of the structure of geometric structures 2 and 3 were 27.5% and 45%, respectively for all physiotherapy students. On average, the geometric structure 1 was rated as the easiest of all three tasks, while the geometric structure 2 was perceived as the most difficult in both groups.

**Figure 4.**
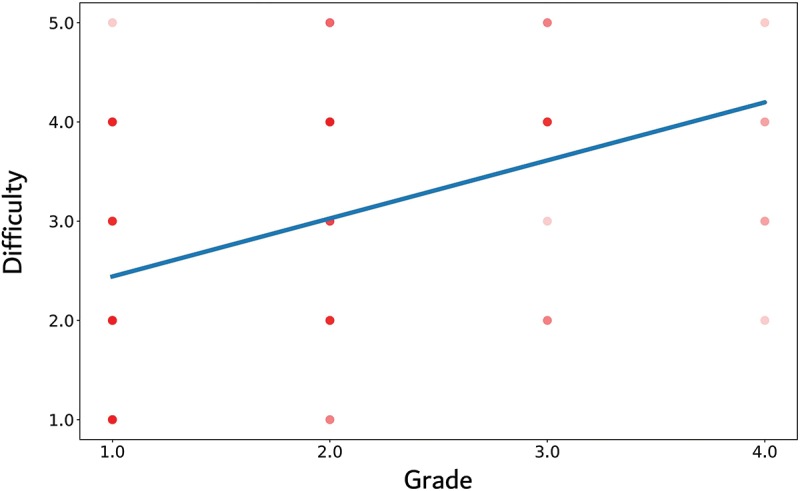
Correlation between the grade of all three geometric structures and the difficulty of the reproduced geometric structure.

The correlation between the grade of all three geometric structures and the difficulty of the reproduced geometric structures is presented in Figure 4. The higher the grade of the reproduced geometric structure was, the more difficult the geometric structure was rated (correlation coefficient *r* = 0.4, r^2^ = 0.16, 95% CI: 0.24–0.54). Approximately 16% of the variance in the difficulty variable can be explained by the grade variable in the linear regression model.

Final-year physiotherapy students spent more time exploring all three geometric structures compared to first-year physiotherapy students. Statistically significant differences were found for geometric structure 2 (*p* = 0.014) and geometric structure 3 (*p* = 0.018) (Table 2). No statistically significant differences were found between the groups in terms of drawing time, accuracy and difficulty of geometric structures (*p* > 0.05) (Table 3).

**Table 2.** Time (average and standard deviations) to explore geometric structures for both groups of students.

Time	First-year group	Final-year group	p-value	ES
Geometric structure 1 (SD) [s]	23.93 (12.30)	30.70 (12.39)	0.0911	−0.55
Geometric structure 2 (SD) [s]	83.10 (37.57)	128.50 (45.30)	0.0014	−1.09
Geometric structure 3 (SD) [s]	55.24 (25.94)	91.42 (40.49)	0.0018	−1.06

*Note: SD – standard deviation, s – seconds, ES – effect size*.

**Table 3.** Time (average and standard deviations) to draw geometric structures for both groups students.

Time	First-year group	Final-year group	p-value
Geometric structure 1 (SD) [s]	8.82 (6.62)	8.35 (4.97)	0.799
Geometric structure 2 (SD) [s]	29.16 (16.44)	35.73 (21.97)	0.290
Geometric structure 3 (SD) [s]	21.84 (14.20)	27.29 (17.06)	0.279

*Note: SD – standard deviation, s – seconds*.

The examiners’ average grades for the reproduced geometric structures for intra-rater agreement are shown in Table 4.

**Table 4.** Average grades (grade a and grade b) (from 1 to 4) and standard deviations of all three geometric structures by both groups of students.

Grade	First-year group	Final-year group	p-value
Grade a of geometric structure 1 (SD)	1.45 (0.94)	1.35 (0.81)	0.721
Grade b of geometric structure 1 (SD)	1.45 (0.94)	1.30 (0.66)	0.563
Grade a of geometric structure 2 (SD)	2.15 (0.75)	1.95 (0.89)	0.445
Grade b of geometric structure 2 (SD)	2.15 (0.75)	1.90 (0.91)	0.348
Grade a of geometric structure 3 (SD)	1.85 (0.75)	1.60 (0.88)	0.339
Grade b of geometric structure 3 (SD)	1.90 (0.72)	1.55 (0.83)	0.161

*Note: SD – standard deviation*.

For the examiner, the intra-rater agreement of the drawing assessment was high (96.7%; Wilcoxon: *t* = 2.5, *p* = 0.32).

## Discussion

The aim of the present study was to compare the palpation skills of first- and final-year physiotherapy students. Our data showed that final-year physiotherapy students spent significantly more time exploring geometric structures 2 and 3, in comparison to their first-year students. This finding may be attributed to the acquired skill of slow palpation developed during their educational program. Geometric structure 1 with the trapezoidal relief, which was rated as the simplest, was correctly reproduced by 77.5% of all physiotherapy students. Similarly, Nascimento et al. [30] found that the geometric structure with the triangular relief as the simplest geometric structure was correctly reproduced by 90% of all participants (*n* = 39). Prior cognition of a common geometric shape may have helped with tactile recognition of the simpler figure [30]. Our participants were more accurate in reproducing the simpler geometric structure compared to the complex ones. When palpating two-dimensional geometric structures with varying degrees of difficulty, more errors are presented when recognizing more complex geometric structures [32]. However, there was no statistically significant difference in accuracy between the groups, but the first-year group received a slightly better grade on average.

The final-year physiotherapy students spent more time exploring structure compared to first-year students. The higher time expenditure could be related to the learned slow palpation of the final-year students, while the lower time expenditure in the first-year students could be related to the lack of experience in palpation and the ambition to complete the task as quickly as possible. However, we can also assume that the final-year students prioritized accuracy improvement over speed and therefore used a different performance strategy than the first-year students, as learning theories report that subjects unconsciously develop different behavioral patterns that increase speed or accuracy to perform a task better [33]. Palpation of different geometric structures requires concentration, spatial awareness and the importance that the participant attaches to the task [34]. Nascimento et al. [30] reported that graduate physiotherapy students spend less time on exploration compared to undergraduate physiotherapy students, which could be related to better palpation skills gained (acquired) during education. In contrast to our results, the main differences between first-year and final-year physiotherapy students in terms of drawing time, accuracy and difficulty of geometric structures were not found. The reason could be the lack of training and experience in palpation of both groups and no ingrained tactile sensation in the brain. Repeated training of a particular skill with rest periods in between is necessary for successful motor skill learning [35]. Daily training leads to anchoring of perceptual ability in somatosensory association areas with activity modulations in partial cortex [34]. Specific and selective changes in the cortex occur only in the second phase of long-term motor skill learning or training [11,36].

The palpation skills of physiotherapy students in our study can be related to the present teaching program and the tactile experiences they received during physiotherapy education. Palpation skills are the competence of physiotherapy students [30], osteopathy students [37], medical students [38] and healthy individuals [12].

This study has certain limitations. Although we performed the evaluation of geometric structures according to the authors’ recommendations [12], the classification was left to the subjective judgment of the rater. Perhaps calculating the total area under the curve between the true shape and the drawn shape would provide a more objective measure of accuracy. The rating scale [12] has not been validated. The fact that only one rater was responsible for scoring the geometric structures and that there was only one week between the second scoring of the drawings could increase the risk of bias and is a major limitation of the study. The next limitation of this study was the small sample size and the varying experience of the final year physiotherapy students. Experience is important not only for the acquisition of palpation skills but also for the reproducibility of clinically relevant tests, such as the examination of trigger points [39], although Sabini et al. [40] found no difference between physicians and medical students in palpating different objects. However, palpation skills depend not only on previous experience [1], but also on individual susceptibility, training, age and disease [11–16]. Another weakness of our study was that the wooden, sunken relief is not comparable to human tissue. Our assessment tool focused on palpating the surface but is certainly not content-specific for palpating living tissue. However, it could be a tool for learning, practicing, measuring and assessing surface palpation skills.

## Conclusions

The level of education and gained experience during laboratory teaching may play an important role in improving the palpation skills of physiotherapy students. Given the importance of palpation skills and the ability to improve these skills with practice, teaching exercises that incorporate the tactile sensitivity test should be included in physiotherapy education programs as a tool to train and assess tactile sensitivity in students. This may help educators develop increased palpation skill, ability, and confidence in their students. Future studies should use a larger sample and compare the results with experienced physical therapists, musicians, and blind people who may have better palpation skills.
